# Iron Oxide Nanoparticle-Based Hyperthermia as a Treatment Option in Various Gastrointestinal Malignancies

**DOI:** 10.3390/nano11113013

**Published:** 2021-11-10

**Authors:** Julian Palzer, Lea Eckstein, Ioana Slabu, Oliver Reisen, Ulf P. Neumann, Anjali A. Roeth

**Affiliations:** 1 Department of General, Visceral and Transplant Surgery, RWTH Aachen University Hospital, 52074 Aachen, Germany; jpalzer@ukaachen.de (J.P.); lea.eckstein@rwth-aachen.de (L.E.); uneumann@ukaachen.de (U.P.N.); 2 Institute of Applied Medical Engineering, Helmholtz Institute Aachen, RWTH Aachen University Hospital, 52074 Aachen, Germany; slabu@ame.rwth-aachen.de (I.S.); oliver.reisen@rwth-aachen.de (O.R.); 3 Department of Surgery, NUTRIM School of Nutrition and Translational Research in Metabolism, Maastricht University, 6229 HX Maastricht, The Netherlands

**Keywords:** iron oxide nanoparticles, magnetic fluid hyperthermia, photothermal therapy, cancer

## Abstract

Iron oxide nanoparticle-based hyperthermia is an emerging field in cancer treatment. The hyperthermia is primarily achieved by two differing methods: magnetic fluid hyperthermia and photothermal therapy. In magnetic fluid hyperthermia, the iron oxide nanoparticles are heated by an alternating magnetic field through Brownian and Néel relaxation. In photothermal therapy, the hyperthermia is mainly generated by absorption of light, thereby converting electromagnetic waves into thermal energy. By use of iron oxide nanoparticles, this effect can be enhanced. Both methods are promising tools in cancer treatment and are, therefore, also explored for gastrointestinal malignancies. Here, we provide an extensive literature research on both therapy options for the most common gastrointestinal malignancies (esophageal, gastric and colorectal cancer, colorectal liver metastases, hepatocellular carcinoma, cholangiocellular carcinoma and pancreatic cancer). As many of these rank in the top ten of cancer-related deaths, novel treatment strategies are urgently needed. This review describes the efforts undertaken in vitro and in vivo.

## 1. Introduction

**Figure 1:**
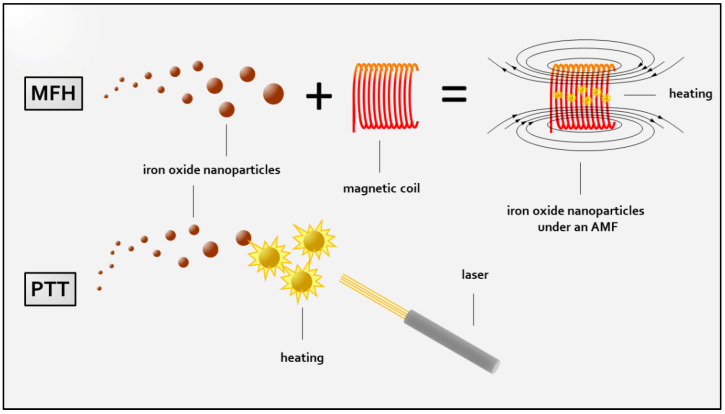
Schematic illustration of basic principles of iron oxide nanoparticle-derived hyperthermia generation. Magnetic fluid hyperthermia (MFH) is achieved by application of an alternating magnetic field (AMF) in the presence of nanoparticles. In photothermal therapy (PTT), heating is accomplished by laser irradiation of nanoparticles through conversion of light energy into thermal. For reasons of simplification, bare nanoparticles are depicted, and nanoparticle modifications are not displayed.

The thriving development of nanomedicine in modern medicine and medical research has opened a limitless landscape of opportunities for alternative treatment strategies in the fight against cancer. Despite the controversial discussion regarding a uniform definition, nanomedicine can be referred to as the “the application of nanotechnology to health” as defined by the European Technology Platform 2005 [1], where nanotechnology in sensu lato refers to the controlled utilization of nanoscale material (<100 nm) [2]. Among the plethora of nanoparticles that have been created and investigated in this context, a particular subgroup, iron oxide nanoparticles (IONs), is widely accepted as a cornerstone due to their strikingly versatile scope of applicability. This includes the use as a contrast agent for MRI imaging in diagnostic [3,4] or theranostic applications [5,6], or even therapeutic hyperthermia treatment [7] due to the magnetic iron oxide core consisting of maghemite (γ-Fe_2_O_3_) or magnetite (Fe_3_O_4_) [8]. Expanding the versatily of ION-based therapy employment of specific surface modifications provide a multimodal therapeutic platform for (targeted) drug delivery [9,10], targeted gene therapy [11] or immunotherapy [12]. Such surface modifications, especially certain coatings of IONs, furthermore, not only enhance their biocompatibility [13] to neglectable a priori cytotoxicity [14] but also reduce biodegradation via the reticuloendothelial system [15] which occurs simoultaneous to the more dominant ways of size-dependent renal and hepatic elimination [16]. Regarding the commonly encountered insufficiency of conventional therapy approaches such as in pancreatic cancer due to common resistance towards current therapy standards [17,18], ION-based hyperthermia has raised expectations to emerge as a novel therapy option by overcoming such drawbacks. ION-based hyperthermia is primarily achieved by two differing methods: magnetic fluid hyperthermia (MFH) and photothermal therapy (PTT). The basic principles of each treatment are captured in Figure 1. Generally, PTT describes the use of light for localized hyperthermia ablation though partial conversion of high-frequency electromagnetic waves into thermal energy by the absorption of light, eventually, attaining promising cell death effects [19]. The effect can be enhanced by the use of exogenous metal nanostructures such as IONs, which potentiate energy transformation by acting as resonance bodies. Additionally, commonly used near-infrared irradiation (NIR) allows for increased in-depth tissue penetration [20]. Among many different nanoparticles suitable for PTT, superparamagnetic iron oxide nanoparticles (SPIONs) are widely regarded as the nanoparticles of choice due to their approval for clinical implementation by the FDA [14]. Magnetic fluid hyperthermia, in contrast, uses an alternating magnetic field to induce heating of SPIONs [21]. The characteristic of superparamagnetism relies on their nanoscale size and leads to immediate loss of internal electromagnetic charge upon termination of an external AMF, thereby preventing agglomeration [22]. In fact, heat generation is achieved by hysteresis loss in terms of Brownian and Néel relaxation of superparamagnetic iron oxide nanoparticles under high frequency (100–500 kHz) AMF exposure [21,23]. Although numerous MFH-suitable nanoparticles have been designed, superparamagnetic iron oxide particles (SPIONs) remain a popular choice [24] in the investigation of the clinical applicability of MFH treatment for multiple cancer entities [25,26], ultimately even obtaining clinical approval by the FDA for the treatment of glioblastoma in 2013 [27]. This vibrant development naturally reached a variety of tumor entities, including gastrointestinal (GI) malignancies. Some of the most common gastrointestinal malignancies include: (1) esophageal cancer, (2) gastric cancer, (3) colorectal cancer, (4) colorectal liver metastasis, primary liver cancer pre-eminently comprising (5) hepatocellular carcinoma (75–85% of all cases) as well as (6) cholangiocellular adenocarcinoma (10–15% of all cases), and (7) pancreatic cancer ranks at the top regarding incidence as well as cancer-related deaths among all cancer entities, in sum, claiming more than 3 million deaths worldwide (in 2020) [28]. When put into perspective, among all cancer cases, some of these cancer entities such as stomach, liver, esophageal and pancreatic cancer count for more or less as many deaths as newly diagnosed cases [28]. The fact that the individual death rates of these malignancies almost match their incidence rates may be considered as a surrogate parameter for the treatment efficiency of these malignancies, herein indicating an urgent need for novel effective strategies in the battle against these cancers. Therefore, elaborate efforts have been made to study the effects of PTT and MFH on these tumor entities and their eligibility regarding their implementation as novel treatment options. These studies comprise a variety of experimental settings. Experiments were performed in vitro, which related to cell culture experiments, and in vivo, referring to animal studies where animals were inoculated with cancer cells, subsequently growing solid tumors before undergoing specific treatment. When the latter is performed using human cancer cells, the resulting tumor is then called a xenograft and can either be localized in the corresponding organ, so-called orthotopic xenograft or at an anatomical position which does not match the tumor origin, then called a heterotopic xenograft. If cancer cells that are derived from the same species are used for this purpose, it is referred to as an allograft. Aiming to provide a descriptive overview over the advances in the development of ION-based hyperthermia (ION-HT) as a novel anti-cancer treatment option for various GI malignancies, we summarized the investigations on MFH and PTT in esophageal, gastric and colorectal cancer, colorectal liver metastasis, hepatocellular carcinoma, cholangiocellular adenocarcinoma and pancreatic cancer.

## 2. Materials and Methods

**Figure 2:**
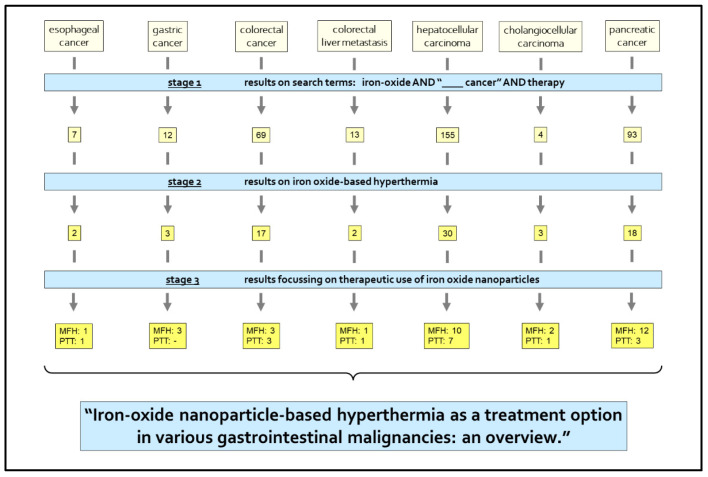
Research process: in stage 1 a first search using the search terms: “iron oxide AND (the respective) cancer AND therapy” was conducted, then the abstracts of these search results were screened. In stage 2, the search was specified to results on iron oxide-based hyperthermia therapy. In the last stage, stage 3, priory elected eligible full papers were examined and their findings summarized in this review. Numbers indicate the number of articles found in each category after each research stage.

Research was performed using pubmed.ncbi.nlm.nih.gov and webofknowledge.com using the MeSH and search terms: *iron oxide, therapy, hyperthermia, esophageal cancer, gastric cancer, colorectal cancer, colorectal liver metastasis, hepatocellular carcinoma, cholangiocellular carcinoma,* *pancreatic cancer* (first time 28 February 2021). Studies focused on therapeutic investigations were included. Initial literature acquisition was performed by a search for “iron oxide AND (the respective cancer entity) AND therapy”. The literature was screened for eligibility and the search modified for specifically hyperthermia treatment. Full papers focusing on therapeutic use of ION-based hyperthermia were reviewed and summarized in this work. The process of this research work is depicted in a flow chart in Figure 2.

## 3. Findings

**Figure 3:**
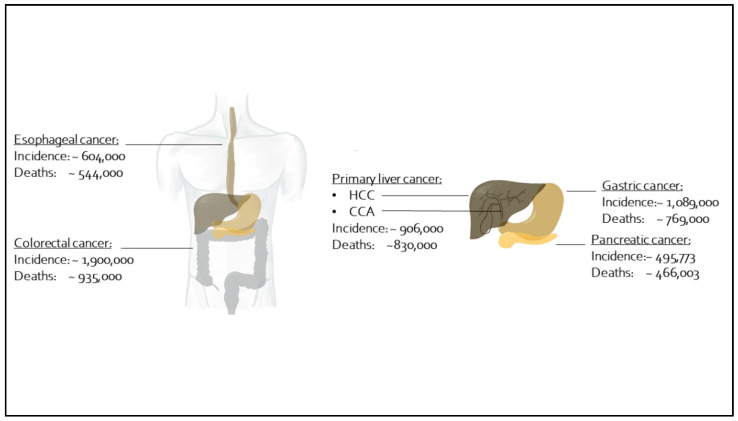
Worldwide incidence and annual death counts in 2020 [28] of selected gastrointestinal malignancies: esophageal cancer, gastric cancer, pancreatic cancer, colorectal cancer, colorectal liver metastasis and primary liver cancer, including hepatocellular carcinoma (HCC) and cholangiocellular adenocarcinoma (CCA).

Figure 3 depicts the seven different gastrointestinal malignancies entities described in this review. In addition, it clarifies the worldwide incidences, and total numbers of deaths due to the respective entity. The initial literature search provided 7 results for esophageal cancer, 12 for gastric cancer, 69 for colorectal cancer, 13 for colorectal liver metastasis, 155 for hepatocellular carcinoma, 4 for cholangiocellular adenocarcinoma and 93 for pancreatic cancer. Eventually, 31 full papers on MFH and 16 full papers on PTT were included in this review. In the following, we describe each entity regarding MFH and PTT therapy with IONs.

### 3.1. Esophageal Cancer

Esophageal cancer (EC) not only represents a common GI malignancy but further plays a significant role amidst all cancer entities, ranking 10th in incidence rate as well as accounting for ~544,000 deaths worldwide in 2020, amounting to 5.5% of all cancer-related deaths [28]. Interestingly, little research has been conducted regarding ION-based hyperthermia, although with the endoluminal localization, the magnetic field traps necessary for AMF generation, might be placed endoscopically easily, thereby making it an excellent entity for MFH.

#### 3.1.1. Esophageal Cancer: MFH

Roeth et al. investigated magnetic trapping of SPIONs at the tumor site of an esophageal adenocarcinoma, with perspective to future peripheral SPION injection and following endoscopically guided MFH treatment in a virtual model. Based on SQUID measurements of magnetic susceptibility of porcine and rat tissue and clinical data analyzing tumor volume and topographical relations, a virtual biophysical model was developed using MATLAB^®^. The enabled simulations eventually demonstrated a potential 8-fold increase of SPION accumulation through ideal magnetic trapping [29]. Herewith, the possibility of tumor therapy with magnetic nanoparticles by use of endoscopically placed magnetic field traps could be demonstrated.

#### 3.1.2. Esophageal Cancer: PTT

The only study on ION-based HT treatment with PTT in esophageal carcinoma was performed by Chu et al. testing different SPIONs derivates and wavelengths with respect to their suitability for PTT prior to in vitro and in vivo evaluation of the anti-cancer effects of PTT. In vitro findings showing marked cell death could be affirmed in a heterotopic xenograft mouse model as repeated NIR of 20 min at 24-h intervals of Fe3O4/(DSPE-PEG-COOH)-injected mice resulted in significant tumor volume reduction [30].

### 3.2. Gastric Cancer

Although recent years have depicted a mildly decreasing mortality for gastric cancer, it is still characterized by an increasing incidence. Therefore, it accounts for high numbers in incidence and death rates [28,31]. Among GI malignancies, gastric cancer appears as one of the leading killers, determining nearly 800,000 deaths in 2020 [28]. Despite promising findings, only a few studies addressing ION-HT, solely on MFH, have been conducted.

#### Gastric Cancer: MFH

The earliest reports on ION-based hyperthermia treatment were published by Yoshida et al. in 2010, evidencing a superiority of combined-dual thermo-chemotherapy using doxorubicin-functionalized magnetite-magnetoliposomes compared with single chemo- or thermotherapy. This was performed in a heterotopic xenograft mouse model by demonstrating prolonged survival and reduced tumor volume following treatment-induced cell death [32]. A follow-up trial investigated the underlying effect of this observation and could relate it to apoptosis as well as necrosis; furthermore, it was associated with TNF-α signaling [33]. Apoptosis refers to controlled cell death following distinct stimuli, whereas necrosis describes chaotic cell collapse upon unbearable cellular damage [34]. In both studies, nanoparticles were injected directly into the tumor. This is a common procedure in experimental settings but it holds the risks of fostering metastasis along the injection site [29]. Intending to circumvent this risk by intravenous injection of targeted nanoparticles, Ruan et al. created fluorescent labelled and SPION-functionalized mesenchymal stem cells and used them in a heterotopic MFC allograft mouse model for targeted MFH treatment [35]. Here, targeting was attributed to tumor homing properties of mesenchymal stem cells which tend to accumulate in tissue with high angiogenesis rates, as overexpressed in malignant tissue. Results showed both efficient nanoparticle accumulation at the tumor side (MRI, fluorescence) and significant inhibition of tumor growth upon MFH treatment at high temperatures ranging around 60 °C, evaluated one week after injection. Adding to the successful targeting of IONs to the tumor, in vivo visualization of this IONs distribution using MRI imaging illustrated the promising potential regarding theranostic use of IONs.

### 3.3. Colorectal Cancer

Culminating in 1.9 million estimated new cases in 2020, colorectal cancer incidence has been on a constant rise. Reasons are, amongst other factors, likely attributed to unhealthy lifestyle choices, including poor physical activity, alcohol and tobacco consumption [36]. With this, colorectal cancer not only ranked third in worldwide incidence in 2020, but further is the second leading cancer death cause after lung carcinoma, with 935,000 colorectal cancer-related deaths in 2020 [28]. This is despite established therapy concepts including surgery in combination with adjuvant chemotherapy for colon cancer and upper rectal cancer or neoadjuvant radiochemotherapy for lower rectal cancer. Given this sheer number and the resulting urge for improvement, nanoparticle-based hyperthermia has found its way into the research for novel treatment strategies in colorectal cancer.

#### 3.3.1. Colorectal Cancer: MFH

Regarding investigations on ION-based MFH treatment of CRC, its effects were first assessed in vitro on Caco-2 cells by Rodríguez-Luccio et al. by comparison of MFH to external (hot water) hyperthermia regarding cytotoxic effects. Here, MFH presented with superior cytotoxicity which appeared in a time-delayed manner peaking at 48 h post-treatment and was most pronounced for a longer treatment period of 2 h [37]. Further investigations contributed by Hardiansyah et al. and Dabaghi et al. tested combined ION-based thermo-chemotherapy against either therapy alone in vitro [38] as well as in vivo [39] in heterotopic xenograft mouse models using the chemotherapeutic doxorubicin and 5-Fluouracil, respectively. Both constituted an additive anti-tumorigenic effect of MFH to the toxicity of the chemotherapeutic agent. Hardiansyah et al. observed significantly increased cytotoxicity of thermo-chemotherapy compared with therapy alone at 24 h after the treatment in a CT-25 mouse colon carcinoma cell culture model, which was primarily attributed to enhanced Doxorubicin release [38]. Interestingly, this superiority of thermo-chemotherapy compared with exclusive chemotherapy appeared to vanish over time as samples treated with either therapy showed almost equally reduced cell survival at 72 h after the individual treatment. In contrast, over the same time period the cytotoxic effects of thermo-chemotherapy remained stronger than such resulting from MFH monotherapy. Dabaghi et al. later demonstrated significant superiority of thermo-chemotherapy compared with 5-Fluouracil monotherapy in vivo in a heterotopic HT 29 xenograft mouse model regarding tumor volume reduction and attenuation of proliferation activity [39]. Interestingly, exclusive MFH treatment also showed significant tumor volume reduction, which was partially attributed to DNA damage in terms of DNA double-strand breaks.

#### 3.3.2. Colorectal Cancer: PTT

Initial investigations addressing PTT of colorectal cancer cells were conducted by Kirui et al. presenting effective in vitro treatment of CRC cells using immune-targeted gold-hybrid nanoparticles. These were also capable of successfully targeting specific colorectal cancer cells as well as acting as MRI contrast agents. PTT effects succumbed power-dependent cellular death pathways: cell death at lower powers were mainly attributed to apoptosis, whereas higher powers primarily resulted in necrosis [40]. By translation of this concept to an in vivo setting using SW112 xenograft mice, the same group found significant tumor growth inhibition upon seven-course PTT treatment which was correlated with marked necrosis of ~65% more in the treatment group [41]. Extending this approach of targeted treatment, Yang et al. employed the chemotherapeutic agent *irinotecan* (a topoisomerase inhibitor) as well as anti-CD133 antibodies targeting CD133, a surface structure overexpressed by cancer stem cells, onto targeted SPIONs to achieve targeted thermo-chemotherapy, which resulted in potent tumor growth inhibition significantly surpassing the effects of either treatment alone [42]. This was in line with preceding in vitro cytotoxicity experiments performed in the same study on three CRC cell lines, which also evidenced the superiority of thermo-chemotherapy.

### 3.4. Colorectal Liver Metastasis

A common problem faced when dealing with colorectal cancer is the high rate of liver metastasis at initial diagnosis which amounts to ~35% [43]. It is due to anatomical conditions that colorectal cancer tends to metastasize into the liver by following portal venous drainage. Although there are several surgical strategies for resectable colorectal liver metastasis, due to cases of extended metastasis, curative resection can still often not be achieved [43]. Hence, liver metastasis still represents a challenge which indicates a clear demand for novel curative treatment approaches.

#### 3.4.1. Colorectal Liver Metastasis: MFH

Facing this issue, Arriortua et al. addressed the feasibility of targeted hybrid magnetic gold particles for MFH treatment of CRC liver metastasis in an in vivo orthotopic CC-531 allograft rat model, eventually demonstrating selective tumor necrosis resulting from AMF application in the presence of previously intra-arterially applied targeted SPIONs [44].

#### 3.4.2. Colorectal Liver Metastasis: PTT

A similar concept of targeted hyperthermia was also tested for PTT in vivo on a CC-531 orthotopic allograft rat model in a similar setting, likewise, including intravascular ION administration. PTT treatment also resulted in potent cell death [45]. Novell, MRI imaging was not only employed to monitor ION accumulation but also postulated to potentially enable treatment optimization in terms of MRI guided adjustment of the irradiation area, hence, supporting precision treatment.

### 3.5. Primary Liver Cancer 

At a high incidence of ~906,000 cases in 2020, primary liver cancer, comprising predominately hepatocellular carcinoma (HCC) but also, and in a far smaller fraction of ~15%, cholangiocellular carcinoma (CCA), holds up for the third highest cancer-related death cases [28]. Viral hepatitis, especially due to the Hepatitis C Virus, alcohol abuse, as well as obesity-related non-alcoholic fat liver disease (NAFLD) are among the risk factors for the development of HCC [46,47,48]. For CCA, risk factors include primary bile duct diseases, alcoholic liver disease, and diabetes mellitus [49]. Liver cancer incidence and death counts have increased from ~841,000 to ~906,000 new cases and ~782,000 to ~830,000 deaths in 2018 and 2020, respectively [28,31]. Facing this trend, ION-based hyperthermia concepts of MFH treatment and PTT have been addressed in the development of novel treatment strategies.

#### 3.5.1. Hepatocellular Carcinoma

##### Hepatocellular Carcinoma: MFH 

Liao et al. found in an in vitro HepG2 cell culture model that one-time SPION-derived MFH treatment accounting for a ~40% cell viability decrease could be enhanced by active targeting using galactosamine by an additional 55% decrease, thereby leaving only 5% viable cells. It was suggested that the cytotoxic effects depends on the overall intracellular iron-content which was significantly higher in the targeted treatment group due to increased cellular uptake [50]. Achieving additional imaging control by X-ray, Attaluri et al. functionalized bionised nano-ferrite (BNF) nanoparticles with Lipidiol, an ethiodised oil, which is a contrast agent in computer tomography imaging and especially used in detection of hepatocellular carcinoma. After intra-arterial administration of BNF-lip, MFH treatment of VX2 allograft rabbits resulted in significant tumor necrosis of 35%. Previous heating potential analysis of BNF-lip and plain BNF particles in a heterotopic HepG2 xenograft mouse model showed superior heating of BNF-lip which was attributed to the formation of larger iron oxide aggregates enabled by the coating [51]. Yang et al. showed efficient targeting of SPION-containing ML, which are SPIONs encapsulated with phospholipids for increased biocompatibility, by modification with anti-CD90-antibodies resulting in improved tumoricidal effects against CD90-positive Huh7 hepatocellular cancer cells, a liver cancer stem cell line, in terms of apoptosis-related cytotoxicity and tumorigenic ability. Interestingly, CD90-negative Huh7 cells and CD90 positive cells showed almost identical a priori thermo-sensitivity towards untargeted MFH. Further transfer to an in vivo model of Huh7 xenograft mice affirmed this potential as targeting generated a two-fold increase in inhibition rate of tumor volume and tumor mass, which was suggested to be apoptosis-related [52].

Testing an alternative inductive heating strategy, Zuchini et al. compared SPION-derived hyperthermia to inductive heating (achieved by exposure of intratumorally placed stainless-steel fine-needles to an AMF) as well as combined treatment in vivo in a heterotopic N1-S1 xenograft rat model [53]. Here, repeated combined treatment of three courses and one-time treatment using novel two-part needles attained a 100% treatment response, defined as a reduction in tumor volume and an increase in survival time [54], exceeding the effects of either therapy alone. At this, apoptotic and necrotic processes were observed. Among all groups, SPION-based MFH treatment accounted for the least pronounced effect with merely 18% treatment response, which was attributed to poor SPION distribution within the tumor. Jeon et al. exploited synergistic effects of MFH and doxorubicin-based chemotherapy achieved by AMF application in the presence of doxorubicin-loaded IONs [55] in a heterotopic luciferase-expressing Hep3B xenograft mouse model. Thermo-chemotherapy achieved higher cell death rates than either therapy alone as well as permanent attenuation of tumor activity. Thereby, thermo-chemotherapy exceeded not only the moderate effects of exclusive doxorubicin therapy but was also capable of overcoming rebound phenomena as observed for exclusive MFH. This effect was associated with increased doxorubicin concentrations at the tumor site arising from prolonged doxorubicin release due to ION accumulation and uptake at the tumor site. The potential of thermosensitive doxorubicin-loaded SPIONs for enhanced drug delivery was already previously investigated by Purushotham et al. [56]. Another contribution to this topic was provided by Wang et al. by a comparison of differently shaped magnetic mesoporous silica nanoparticles which were either sphere-like or rod-like shaped for their potential regarding MFH augmented with suicide gene therapy [57]. Suicide gene therapy refers to the introduction of a gene enabling the conversion of a prodrug into a toxic agent, which, eventually, causes cell death selectively in the tumor cell. Experiments were conducted in vitro in a HepG2 cell culture model and in vivo in a Hep2G cell culture model with peripheral intravenous administration of IONs with subsequent targeting achieved by using an external magnetic field directed at the tumor site. Data obtained in vitro provided various observations, including superiority of untargeted MFH-augmented suicide gene therapy compared with untargeted exclusive gene therapy (this applied to both particle shapes). Furthermore, targeted combined therapy using rod-like-shaped nanoparticles demonstrated superiority compared with identical therapy using sphere-like-shaped nanoparticles, untargeted combined therapy using rod-like-shaped nanoparticles and untargeted exclusive gene therapy. This was in coherence with previously demonstrated enhanced uptake, heating profile and drug-release of rod-like-shaped nanoparticles in comparison with sphere-like-shaped nanoparticles. Ensuing in vivo experiments affirmed the potential of rod-like-shaped nanoparticles as targeted and combined therapy accounted for the most prominent tumor growth inhibition, as well as relative tumor mass reduction among all tested approaches. The latest study on ION-based thermo-chemotherapy and combined theranostics is an in vivo study contributed by Chan et al. [58]. Using the chemotherapeutic agent mitoxantrone, a topoisomerase inhibitor, in an orthotopic Mahlavu and SKHep1 xenograft mouse model Chan et al. demonstrated impressive theranostic feasibility as in vivo MRI imaging was employed to monitor tumor development upon MFH, chemotherapy and combined thermo-chemotherapy. Here, tumor volume reduction appeared most prominent for thermo-chemotherapy, which was later confirmed by postmortem tumor tissue analysis regarding size and weight of the tumors. Furthermore, mice treated with thermo-chemotherapy survived longer than animals treated with single therapy of either kind.

##### Hepatocellular Carcinoma: PTT

Testing citrate-coated SPIONs functionalized with HCC cell targeting anti-glypican-3-antibodies for their potential as photothermal converters in PTT-based theranostics, Li et al. demonstrated dose-dependent cytotoxicity [59]. MRI imaging of efficient targeting and uptake of HepG2 cells by anti-glypican-3-antibody modification demonstrated successful particle distribution in the tumor. Liu et al. used novel targeted particles, SPIONs modified with clusters of copper sulfide particles due to their potential as photothermal agents [60]. In continuation of the previous study, these in vivo investigations affirmed the cytotoxic potential of PTT in HCC. Furthermore, MRI control of intravenously applied IONs evidenced targeting success. Another study led by Li et al. then proposed the concept of minimally invasive laparoscopic-assisted PTT of deep tumors, exploiting beneficial accumulation effects of peripherally administered PEGylated SPIONs through enhanced permeability and retention effects in an orthotopic HepG2 xenograft mouse model [61]. Apart from faster recovery upon treatment, laparoscopic-assisted PTT resulted in the most prominent tumor suppression performance objectified by tumor volume as well as molecular evaluation of necrosis and apoptosis, alongside significantly prolonged survival when compared with conventional anti-neoplastic and surgical approaches. PTT experiments frequently incorporated chemotherapeutic agents and targeting. Liu et al. introduced PTT-assisted trans-arterial chemoembolization (TACE) using doxorubicin [62]. Increased agent accumulation was achieved by embolization, using common embolisate lipodiol as well as pH-related trapping eventuating from a poly-dopamine (PDA) coating. PDA simultaneously acted as a photosensitizer in PTT. In vivo experiments in an orthotopic N1S1 hepatoma rat model showed larger tumor inhibition rates in terms of tumor volume reduction, necrosis and apoptosis induction and proliferation impairment of combined therapy compared with exclusive doxorubicin or ION-enhanced doxorubicin treatment. The same group later demonstrated that such effects could be intensified by pretreatment with an injection of a vascular disruptor called combretastatin A4-phosphate [63]. Huang et al. demonstrated that the combination of therapies with DOX-loaded microspheres containing PDA-functionalized SPIONs improved drug release when a certain temperature threshold was surpassed [64]. Strikingly, PTT/TACE therapy was shown to overcome chemoresistance in HCC cells in vitro by using regular HepG2 cells and chemo-resistant HepG2/ADR cells. CT imaging evaluation of combined therapy efficacy in vivo in an orthotopic VX2 rabbit model demonstrated impressive treatment response exceeding the treatment success stated in the current literature for onetime or multiple TACE treatments. Histopathological analysis demonstrated high necrotic rates as well as complete response rates in 37.5% of the cases. Affirming the potential of combined thermo-chemotherapy using doxorubicin and PDA-SPION, Shu et al. observed greater in vitro cytotoxicity of such thermo-chemotherapy compared with either therapy alone [65]. In vivo experiments on a heterotopic HepG xenograft model as combined therapy likewise accounted for better treatment response, since combined therapy resulted in noteworthy tumor volume reduction, whereas exclusive chemo- or photothermal therapy solely attenuated tumor growth.

### 3.5.2. Cholangiocellular Adenocarcinoma 

So far, to our knowledge, only one study has addressed the feasibility of ION-assisted hyperthermia treatment within the context of cholangiocellular adenocarcinoma [66]. Specifically, hybrid composition of ultrasmall gold nanoparticles and iron oxide nanoflowers (GIONF) chosen for their promising heating properties were used for in vivo investigations on cancer-associated fibroblasts involved in cancer-related desmoplasia, particularly prominent in cholangiocellular adenocarcinoma. PTT, and, especially, repeated three-course PTT, resulted in a reduction in tumor stiffness and an induction of tumor regression due to hyperthermia-induced decay of particularly cancer-associated fibroblasts. Nonetheless, there are studies which use ION-assisted hyperthermia treatment for cholangiocellular carcinoma in other ways than MFH or PTT. Mues et al. incorparted ION in a polymer hybrid stent to enable hyperthermia treatment of hollow organ tumors [67,68]. Explanted porcine bile duct served as example of choice in this feasibility study for future applications in cholangiocellular adenocarcinoma.

### 3.6. Pancreatic Cancer

With an overall 5-year survival of approximately 8% [69] and claiming nearly as many deaths (466,000) as new cases (496,000) [28], pancreatic cancer accounts for a particular miserable prognosis, in part affiliated with the progressed tumor stages at first diagnosis. At this point, only a mere 16% of the patients’ collective qualify for curative surgery [70], which up to this date represents the single curative treatment option, particularly due to the commonly found resistance to chemotherapy in pancreatic cancer [18]. Motivated by this unsatisfactory state, efforts towards the establishment of novel treatment approaches have steadily increased over the course of the past few years, eventually imposing promising expectations on ION-based hyperthermia treatment. In the center of this development clearly stands MFH, whereas studies addressing PTT present a minority.

#### 3.6.1. Pancreatic Cancer: MFH

Despite the wide range of issues addressed by investigations on SPION-based MFH treatment, and the great variety of experimental set-ups and end points, MFH has consistently been accredited with beneficial tumoricidal effects in the treatment of pancreatic cancer.

In general, MFH´s effects were assessed in different approaches that may comprehensively be categorized in two major groups:

(1) Investigations on MFH monotherapy (Pancreatic Cancer: MFH Monotherapy).
(2) Investigations on the combination of MFH with additional therapeutic strategies in dual-therapy approaches (Pancreatic Cancer: MFH in Dual Therapy Approaches).

##### Pancreatic Cancer: MFH Monotherapy

Naturally, investigations on MFH addressed multiple issues in altering settings. Fundamental was the observation of superiority of MFH over external hyperthermia regarding short term cytotoxicity attributed to increased levels of apoptosis, necrosis and reduction in proliferation activity, as well as long-term cytotoxicity in terms of attenuation of cell reproducibility, so-called clonogenic potential, at lower temperatures and shorter heat exposure, respectively [71,72]. Here, in vitro studies by Engelmann et al. revealed significant long-term cytotoxicity in terms of reduced clonogenic potential of Mia PaCa-2 pancreatic cancer cells upon MFH treatment, which is the ability of cell reproduction [71]. Investigation of the uptake kinetics of magnetoliposomes into the pancreatic cancer cell lines Mia PaCa-2 and BxPC-3 by Slabu et al. led to a mathematical model with the possibility to analyze the specific internalization mechanism for each particle-type [73], which influences the cytotoxic effect to a large extent. In vivo investigations demonstrated beneficial tumoricidal effects as well. In fact, single MFH treatment of xenograft mice carrying heterotopic Mia PaCa-2 tumors conducted by Attaluri et al. demonstrated successful attenuation of tumor growth and prolonged survival [74]. They employed computational analysis of differently pulsed AMFs to optimize heat deposition and thermal dosage. Hereby, they successfully reduced AMF cycles while maintaining treatment efficiency through elongation of the treatment duration. Similar findings were obtained for sequential treatment of multiple MFH sessions. Here, the earliest data obtained in a heterotopic Pan02 allograft mouse model resembling pancreatic cancer peritoneal metastasis evidenced the treatment efficacy of MFH as survival increased by 31% upon sequential three-times MFH treatment [75]. Furthermore, sequential MFH treatment of heterotopic BxPC-3 xenograft mice as conducted by Kossatz et al. showed significant tumor growth attenuation with time-delayed tumor regrowth, leading to nearly stable tumor volumes [76], whereas similar treatment of heterotopic PANC-1 xenograft mice as performed by Ludwig et al. led to markedly reduced tumor volumes [72]. In extension, other investigations were employed to investigate specific aspects of MFH-derived cytotoxicity. Here, in vitro studies by Engelmann on Mia PaCa-2 cells testing for specific effects arising from altering ION localization with respect to the cell found that exclusively intracellularly located MFH could induce significant long-term cytotoxicity in cancer cells but not in murine fibroblasts. This effect was in part attributed to so-called nanoheating, which accounts for macroscopically imperceptible heating of the immediate ION environment [71,77]. This approach was later supplemented by the observation of a marked dominance of exclusively extracellularly MFH-derived short- as well as long-term cytotoxicity compared with intra- or intra- and extracellularly located ION [78]. Intending to expand the spectra of application for MFH, in the same study, extracellular MFH was further successfully applied on patient-derived pancreatic cancer organoids, three-dimensional in vitro cell structures, hereby affirming the 2D-cell culture previously observed in short-term cytotoxicity. Earlier, Phieler et al. presented the successful treatment of three-dimensional cell line-derived spheroids, not only demonstrating the efficacy of MFH on pancreatic cancer cells, but also on pancreatic cancer-related desmoplastic tissue [79].

##### Pancreatic Cancer: MFH in Dual Therapy Approaches

Whereas MFH is predominantly combined with chemotherapy and other molecular agents, one study employed hadron therapy, which refers to carbon ion/photon irradiation in addition to MFH [80]. Acknowledging the high rates of resistance to most chemotherapeutic agents, and gemcitabine (GEM) being the gold standard chemotherapeutic agent in pancreatic cancer [18], GEM was the chemotherapeutic agent of choice in all approaches along with additional augmentations. As indicated, most studies encapsulated additional therapeutic agents such as anti-Her-2-antobodies or anti-EGFR-antibodies, capable of targeting overexpressing cancer cells while simultaneously holding intrinsic tumoricidal power [81,82]. Further additions to the particles were the apoptosis inducing pseudo-peptide N6L [83] and curcumin, an agent long known for its anti-cancer effects [84]. Among the trials dedicated to this topic two in vitro studies led by Balasubramanian et al. and Wang et al. were performed exclusively. Both constituted superiority of combined thermo-chemotherapy compared with exclusive MFH treatment [84] or chemotherapeutic [82] treatment regarding cell viability which Wang et al. further showed to be apoptosis related [82]. Data obtained in vivo in a heterotopic Mia PaCa-2 xenograft mouse model by Jaidev et al. later demonstrated reduced tumor growth upon thermo-chemotherapy treatment using GEM and anti-HER-2 antibodies to the extent of size-wise stable disease [81]. The combination of MFH with GEM and N6L exceeded these effects by provoking a marked reduction in tumor volume compared with the initial size, as demonstrated in a heterotopic BxPC-3 xenograft mouse model by Sanhaji et al. [83]. In both in vivo studies, exclusive MFH treatment accounted for minor, insignificant anti-tumor effects notably less pronounced than with combined therapy. The documented increase in tumoricidal effects was mainly attributed to synergistic effects of heating and drug effects [81,84], possibly arising from attenuated DNA repair upon hyperthermia treatment [83], as well as a heat-triggered increase in drug release [84].

#### 3.6.2. Pancreatic Cancer: PTT

Investigations on PTT treatment represented a minority among the studies addressing ION-based hyperthermia treatment of pancreatic cancer species. Nevertheless, evaluation of PTT effects in vitro using hybrid iron oxide core gold-shell nanoparticles of rather high a priori toxicity showed a promising potential of 40–50% additional cell killing on top of the particles’ cytotoxicity in a study provided by Guo et al. [85]. Later, this potential was substantiated by in vitro demonstration of power and concentration-dependent cytotoxicity of PTT treatment using SPION-modified graphene oxides. In addition, in vivo investigations contributed by this study proved that PTT treatment of metastatic lymph nodes resulted in significant lymph node volume reduction due to histopathologically evidenced cell death [86]. The before in the context of MFH mentioned study by Balasubramanian et al. also incorporated testing of PTT in vitro. Here, positive anti-tumoral synergy of additional simultaneous PTT was suggested which was considered to be of multifactorial genesis, including thermically increased drug-release, PTT heating effects as well as synergistic effects arising from the combination of both mechanisms [84].

## 4. Conclusions

Many efforts have been undertaken to explore iron oxide nanoparticle-based hyperthermia as a treatment option in all gastrointestinal malignancies. These include predominantly treatment with MFH as well as use for enhancing PTT effects. Most studies focus on colorectal cancer, hepatocellular carcinoma and pancreatic cancer. MFH as well as PTT demonstrate very promising features in tumor treatment. Nonetheless, most studies are performed on in vitro and some in in vivo animal models. Furthermore, despite the wide range of investigations, some challenges remain unsolved such as the limited penetration depth of PTT. In addition, there are technical challenges regarding the employment of magnetic coils that generate the magnetic field in MFH implementation in patients. For the clinical application, the precise scheme for the thermo-chemotherapy needs to be addressed, e.g., duration and repetition. Future studies should, therefore, try to transfer the positive findings obtained in vitro and in vivo into patients.

## Data Availability

Not applicable.
